# Research hotspots and frontiers of neuromodulation techniques in disorders of consciousness: a bibliometric analysis

**DOI:** 10.3389/fnins.2023.1343471

**Published:** 2024-01-08

**Authors:** Bilian Guo, Qiong Han, Jun Ni, Zhipeng Yan

**Affiliations:** ^1^Department of Rehabilitation Medicine, First Affiliated Hospital of Fujian Medical University, Fuzhou, China; ^2^Department of Rehabilitation Medicine, National Regional Medical Center, Binhai Campus of the First Affiliated Hospital, Fujian Medical University, Fuzhou, China

**Keywords:** disorders of consciousness, neuromodulation techniques, bibliometrics, Web of Science, CiteSpace

## Abstract

**Background:**

The characteristics of disorders of consciousness (DOC) are changes in arousal and/or awareness caused by severe brain injuries. To date, the management of DOC patients remains a complex and challenging task, and neuromodulation techniques offer a promising solution. However, a bibliometric analysis focusing on neuromodulation techniques in DOC is currently absent. The aim of this study is to provide a bibliometric visualization analysis to investigate the research hotspots and frontiers in the field of neuromodulation techniques in DOC from 2012 to 2022.

**Methods:**

The publications were collected and retrieved from the Web of Science (WoS) from 2012 to 2022. CiteSpace and Microsoft Excel were utilized perform the first global bibliographic analysis of the literature related to neuromodulation techniques for DOC.

**Results:**

The analysis included a total of 338 publications. From 2012 to 2022, a consistent yet irregular increase in the number of articles published on neuromodulation techniques in DOC was observed. Frontiers in Neurology published the highest number of papers (*n* = 16). Neurosciences represented the main research hotspot category (*n* = 170). The most prolific country, institution, and author were the USA (*n* = 105), the University of Liege (*n* = 41), and Laureys Steven (*n* = 38), respectively. An analysis of keywords revealed that UWS/VS, MCS, and TMS constituted the primary research trends and focal points within this domain.

**Conclusion:**

This bibliometric study sheds light on the current progress and emerging trends of neuromodulation techniques in DOC from 2012 to 2022. The focal topics in this domain encompass the precise diagnosis of consciousness levels in patients suffering from DOC and the pursuit of efficacious neuromodulation-based evaluation and treatment protocols for such patients.

## Introduction

Consciousness denotes an individual’s awareness and confirmation of their environment and their own existence (Giacino et al., 2018). As two integral components of consciousness, awareness and wakefulness are closely intertwined. The former refers to the activation of the brain, while the latter signifies the perception of the environment and/or oneself (Bernat, 2010). Disorders of consciousness (DOC) are induced by a myriad of pathological conditions, including respiratory and cardiac arrest, traumatic brain injury (TBI), cerebral vascular accidents, gross metabolic disorders, brain diseases, infections, drug abuse, as well as other severe neurological insults. Within this context, consciousness is perturbed by alterations in arousal and awareness, which are structurally or functionally attributed to damages in the ascending reticular formation or rostral midbrain, or extensive lesions of the cerebral hemispheres. Clinically, these perturbations manifest as coma, unresponsive wakefulness syndrome (UWS), previously known as a vegetative state (VS), and minimally conscious state (MCS) (Giacino et al., 2014; Zheng et al., 2023).

Accurate differential diagnosis is not only crucial for the clinical management of DOC patients, but also promotes therapeutic approaches related to functional outcomes. Coma is a medical condition that results in patients being unresponsive and unaware of their environment (Teasdale and Jennett, 1974). When patients begin to open their eyes but display only reflex movements, they are diagnosed with UWS/VS (Laureys et al., 2010). Once patients recover from this state and exhibit fluctuating but reproducible signs of consciousness, they enter the MCS (Giacino et al., 2002). Using advanced neuroimaging and electrophysiological techniques, we can gain a deeper understanding of the biological mechanisms of consciousness recovery and identify well-preserved brain networks in seemingly unresponsive patients, bringing hope for more accurate diagnosis and prognosis (Gosseries et al., 2014; Escrichs et al., 2022). However, existing treatment standards cannot provide guidance for clinical decision-making in such patients, often resulting in inconsistent, inaccurate, and inappropriate interventions (Schnakers and Monti, 2017; Edlow et al., 2020). Therefore, novel therapeutic methods are required.

There is considerable evidence that neuromodulation techniques are emerging as a promising new approach in the treatment of DOC patients in trials, as they regulate neural circuits that mediate arousal and wakefulness (Ragazzoni et al., 2017; Xia et al., 2018). In terms of neuromodulation therapy, it can be further categorized into invasive neuromodulation therapy (INT) and non-invasive neuromodulation therapy (NINT). On the one hand, INT usually involves implanting electrodes or other invasive methods to apply electrical pulses to specific parts of the central or peripheral nervous system, including deep brain stimulation (DBS) (Rezaei Haddad et al., 2019), spinal cord stimulation (SCS) (Yang et al., 2022), and surgery vagus nerve stimulation (sVNS) (Corazzol et al., 2017). On the other hand, NINT can deliver electrical or magnetic stimulation to the brain without surgery. Among non-invasive methods, transcranial direct current stimulation (tDCS) (Zaninotto et al., 2019), transcranial magnetic stimulation (TMS) (O’Neal et al., 2021), Transcutaneous auricular vagus nerve stimulation (taVNS) (Hakon et al., 2020), and median nerve stimulation (MNS) (Feller et al., 2021) have been widely adopted and used in clinical samples. In a series of therapeutic methods for promoting rehabilitation, neuromodulation technology has a direct or indirect regulatory effect on functional connectivity between different brain regions by regulating cortical excitability and neural plasticity (Feng et al., 2020; Liu et al., 2023). Over the past decade, the observed surge in publications clearly indicates that neuromodulation technology has been established as valuable tools for studying DOC.

Here, the objective of this work is to conduct a bibliometric analysis of the scientific production devoted to neuromodulation techniques in DOC using CiteSpace, in an attempt to discover the current research status, prominent areas of investigation, and main trends of researches concerning this group. Although such analysis does not answer any specific research question, it should have been carried out for several reasons such as to (1) help researchers and stakeholders to understand a comprehensive description of scientific knowledge including publication, journals, references, research countries, institutions, authors, and keywords etc.; (2) reveal the current research cooperation models and potential new intersections to promote interdisciplinary research and innovation; (3) identify research gaps and propose future research directions to explore the role of neuromodulation techniques in DOC.

## Materials and methods

### Data source and search strategy

The wide coverage of the WoS Core Collection (WoSCC) database, which provides rich citation analysis tools, indicators, and powerful visualization capabilities, is the most commonly used database for bibliometric analysis (Birkle et al., 2020). Previous studies have convincingly demonstrated the efficacy of bibliometric analysis conducted on the WoSCC database (Wang and Maniruzzaman, 2022; Li et al., 2023). By employing this tool, we set out to retrieve relevant literature published between January 1, 2012, and December 31, 2022. The main topics of data retrieval were “neuromodulation techniques” and “disorders of consciousness.” Therefore, the search terms were as follows: TS = (“neuromodulation techniques” OR “deep brain stimulation” OR DBS OR “Spinal cord stimulation” OR SCS OR “transcranial direct current stimulation” OR tDCS OR “transcranial magnetic stimulation” OR TMS OR “vagus nerve stimulation” OR VNS OR “median nerve stimulation” OR MNS) AND TS = (“disorders of consciousness” OR “disturbance of consciousness” OR “consciousness disorder” OR consciousness).

### Inclusion and exclusion criteria

**Figure 1:**
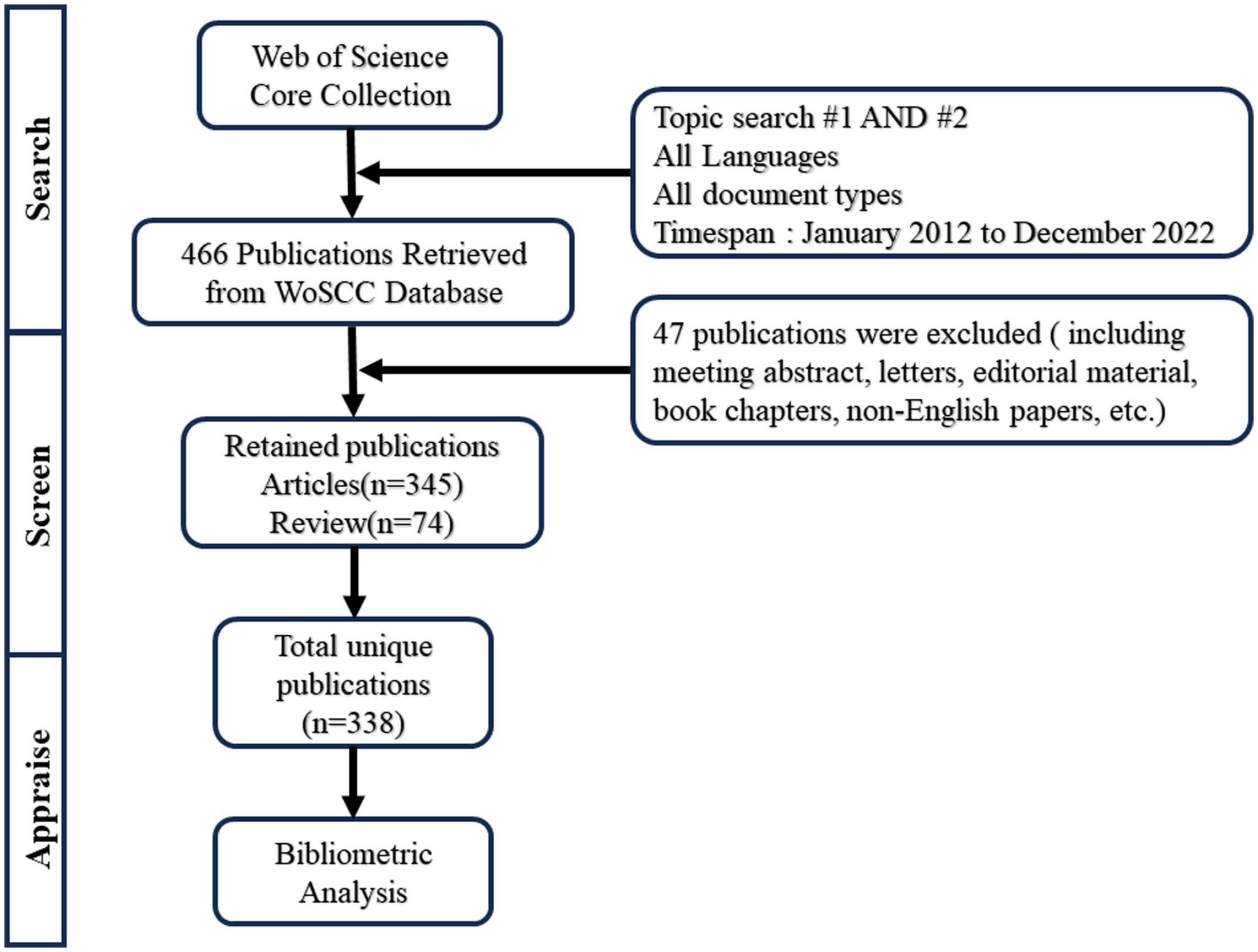
Flowchart of the literature screen. Topic search #1 = (neuromodulation techniques OR deep brain stimulation OR DBS OR Spinal cord stimulation OR SCS OR transcranial direct current stimulation OR tDCS OR transcranial magnetic stimulation OR TMS OR vagus nerve stimulation OR VNS OR median nerve stimulation OR MNS); topic search #2 = (disorders of consciousness OR consciousness disorder OR disturbance of consciousness OR consciousness).

After screening the titles and abstracts, we selected studies that utilized neuromodulation techniques for treating disorders of consciousness. The document type was only limited to articles and reviews. Other irrelevant literature was excluded, including meeting abstracts, letters, editorial material, book chapters, non-English papers, etc. Additionally, the duplicated articles have been eliminated. Before analysis, two researchers independently screened the data. Any discrepancies were discussed and resolved by a third reviewer. The flowchart of the inclusion criteria is presented in Figure 1. Finally, a total of 338 records were retrieved for the purpose of conducting a bibliometric analysis.

### Bibliometric analysis software tools

Data screened from the Web of Science were analyzed using CiteSpace (version: 6.2.R4; Drexel University, USA) and Microsoft Excel 2019. The parameters for CiteSpace were set with a “Time Sliding” value of 1 year, and the node type was selected based on the analysis.

In this study, CiteSpace was used to analyze the dual-map overlap of journals, cluster view, burst detection of cited literature, and centrality of country, institution, and author. The dual-map overlay visualization presents a graphical representation of how articles are distributed, how citation patterns are evolving over time, and how the center of gravity is shifting in different disciplines. The distributions consist of citing journals positioned on the left and cited journals positioned on the right. The citation line represents the context of the citation. The Z-score and F-score provide more consistent and robust trajectory maps, enabling the identification of significant citation paths in the dual-map. Cluster view is conducted on the generated map, where each cluster is identified through citations that include the title, keywords, and subject headings from the abstract of the citing reference. The purpose of burst detection is to identify significant changes in citation numbers over a given period, making it useful for tracking keyword trends. The index of betweenness centrality is used to determine the significance of nodes in a network.

Microsoft Excel 2019 was employed to display publication features and analyze annual publication and citation trends. The data were extracted from Science Citation Index-Expanded of the WoSCC Database, formatted as annual publications, citations, subject categories, countries, institutions, authors, and journals. Besides, the 2022 impact factor (IF), Hirsch index (H-index) and Journal citation reports (JCR) division of journals were also included. IF gages the rank or significance of a journal by calculating the mean number of citations accorded to its selected papers in a given year. The H-index is a metric that measures the scientific impact of an author/country by considering the minimum number of papers (H) that must be published, with each paper receiving at least H citations. JCR is an indexing and evaluation tool that assesses the quality and impact of journals based on their citation analysis data.

## Results

### Analysis of publication outputs

**Figure 2:**
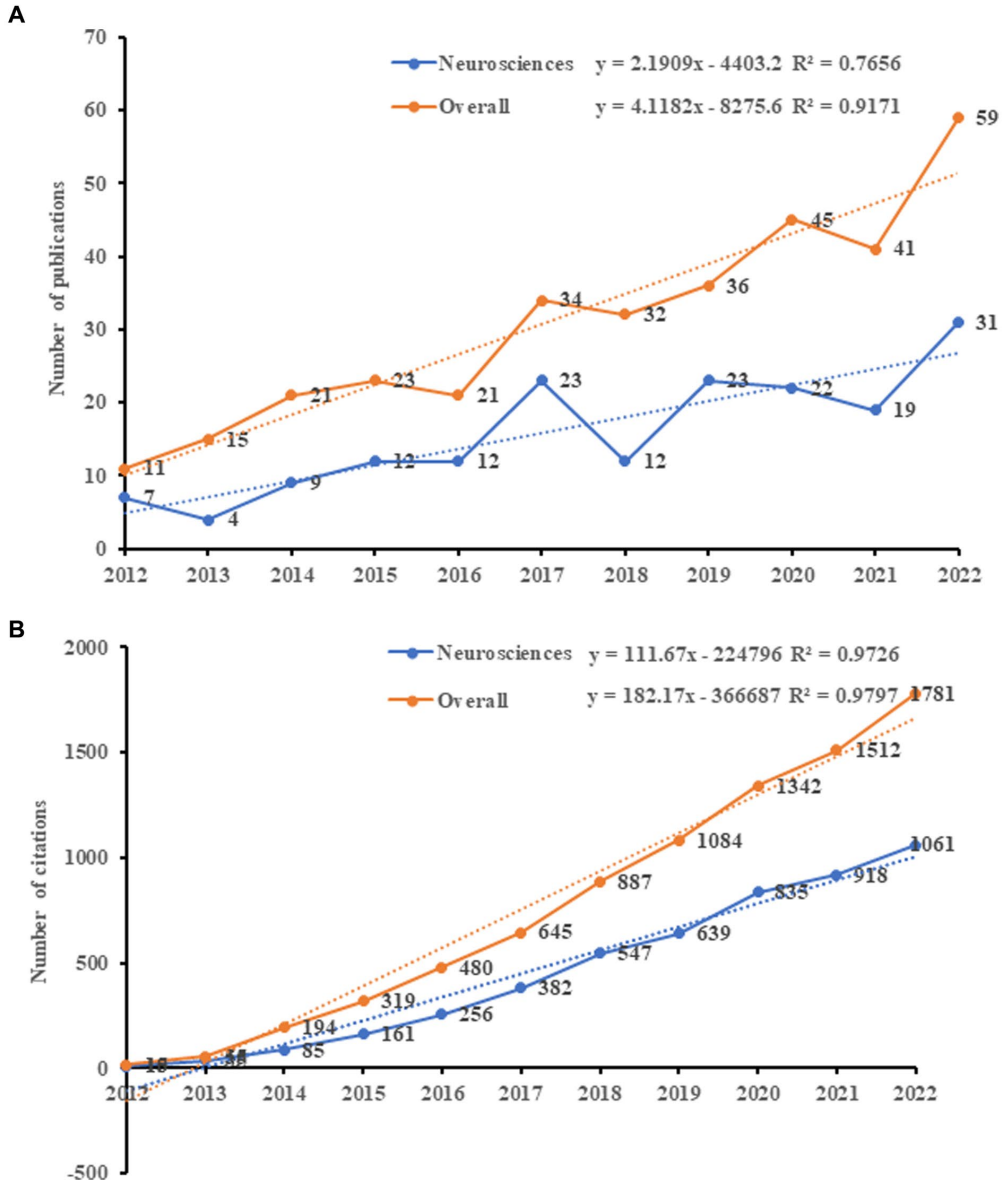
Analysis of publications and citations. Orange represents the overall trend in the broader field, and blue represents the trend in Neurosciences. **(A)** The number of annual publications related to neuromodulation techniques for DOC from 2012 to 2022. **(B)** The number of annual citations related to neuromodulation techniques for DOC from 2012 to 2022.

From 2012 to 2022, a total of 338 articles were analyzed, including 277 articles (81.95%) and 61 reviews (18.05%). Figure 2 illustrates the distribution of annual publications and citations related to neuromodulation techniques in DOC research. We have identified two trend lines, represented by orange and blue. The orange line signifies the overall trend in the broader field, while the blue line indicates the trend in Neurosciences. In terms of annual publications (Figure 2A), the overall trend in the field has undergone two distinct phases over the past decade. Initially, there was a gradual increase in publication numbers between 2012 and 2015, followed by an acceleration leading to a peak between 2016 and 2022, exhibiting a consistent upward trajectory year on year. However, the growth rate in Neurosciences was slower compared to the overall trend, attributed to the less reliable exponential growth model (R2 = 0.7656). Figure 2B illustrates the annual citation distribution of the studies included. The overall trend of citation counts has steadily risen, increasing from 15 in 2012 to 1781 in 2022. Additionally, the growth in Neurosciences aligned with the overall growth. By applying an exponential growth model to evaluate the correlation between citation counts and publication years, the results suggest that the model is consistent with the trend of annual citation volumes (Orange: R2 = 0.9797; Blue: R2 = 0.9726).

**Figure 3:**
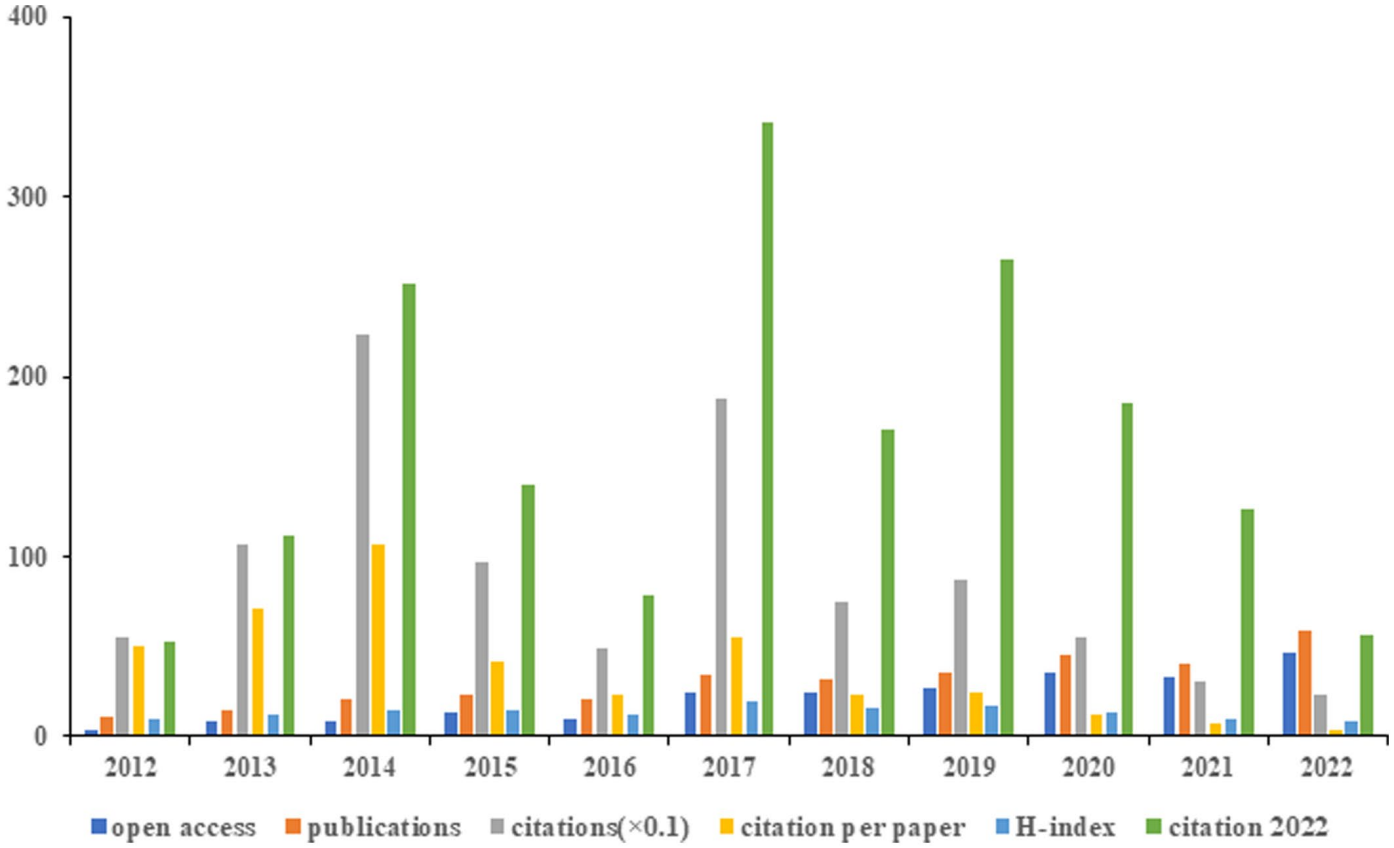
The annual number of open-access articles, publications, citations (×0.1), citations per paper, H-index, and citations in 2022 for each year.

According to Figure 3, it was observed that the highest average number of citation per paper (*n* = 106.38), and citations (*n* = 2,234) were recorded in 2014. The highest H-index (*n* = 19) was observed in 2017. Furthermore, the largest number of published articles (*n* = 59) and open access (*n* = 47) occurred in 2022.

### Analysis of authoritative journals

The 338 studies that were included in this analysis were published across 176 different academic journals. Table 1 displays the details of the top 10 journals. Frontiers in Neurology published the most number of papers (*n* = 16), and the highest open access (*n* = 16), followed by Brain Sciences (*n* = 13), and Frontiers in Neuroscience (*n* = 13). In terms of cited frequency, Clinical Neurophysiology ranked first (*n* = 2,303), followed by PLOS ONE (*n* = 396) and Brain Stimulation (*n* = 361). Brain Stimulation also presented with the highest Impact Factor (IF 2022 = 7.7). Frontiers in Neurology and Brain Stimulation had the highest H-index (*n* = 10).

**Table 1:** The top 10 paper journals based on the number of publications.

Journals	Papers	Citations (WoS)	Citation per Paper	Open Access	Wos Categories	IF (2022)	Quartile of JCR	H-index
Frontiers in Neurology	16	257	16.06	16	Clinical Neurology; Neurosciences	3.4	Q2; Q2	10
Brain Sciences	13	94	7.23	13	Neurosciences	3.3	Q3	6
Frontiers in Neuroscience	13	186	14.31	13	Neurosciences	4.3	Q2	7
Brain Stimulation	12	361	30.08	7	Clinical Neurology; Neurosciences	7.7	Q1; Q1	10
Scientific Reports	10	194	19.4	10	Multidisciplinary Sciences	4.6	Q2	7
Clinical Neurophysiology	7	2,303	329	4	Clinical Neurology; Neurosciences	4.7	Q1; Q2	6
Frontiers in Human Neuroscience	7	277	39.57	7	Neurosciences; Psychology	2.9	Q3; Q2	6
PLOS ONE	7	396	56.57	7	Multidisciplinary Sciences	3.7	Q2	6
Brain Injury	6	145	24.17	3	Neurosciences; Rehabilitation	1.9	Q4; Q3;Q2	6
Cerebral Cortex	5	93	18.6	4	Neurosciences	3.7	Q2	4

WoS, Web of Science; IF, Impact Factor; JCR, Journal Citation Reports.

**Figure 4:**
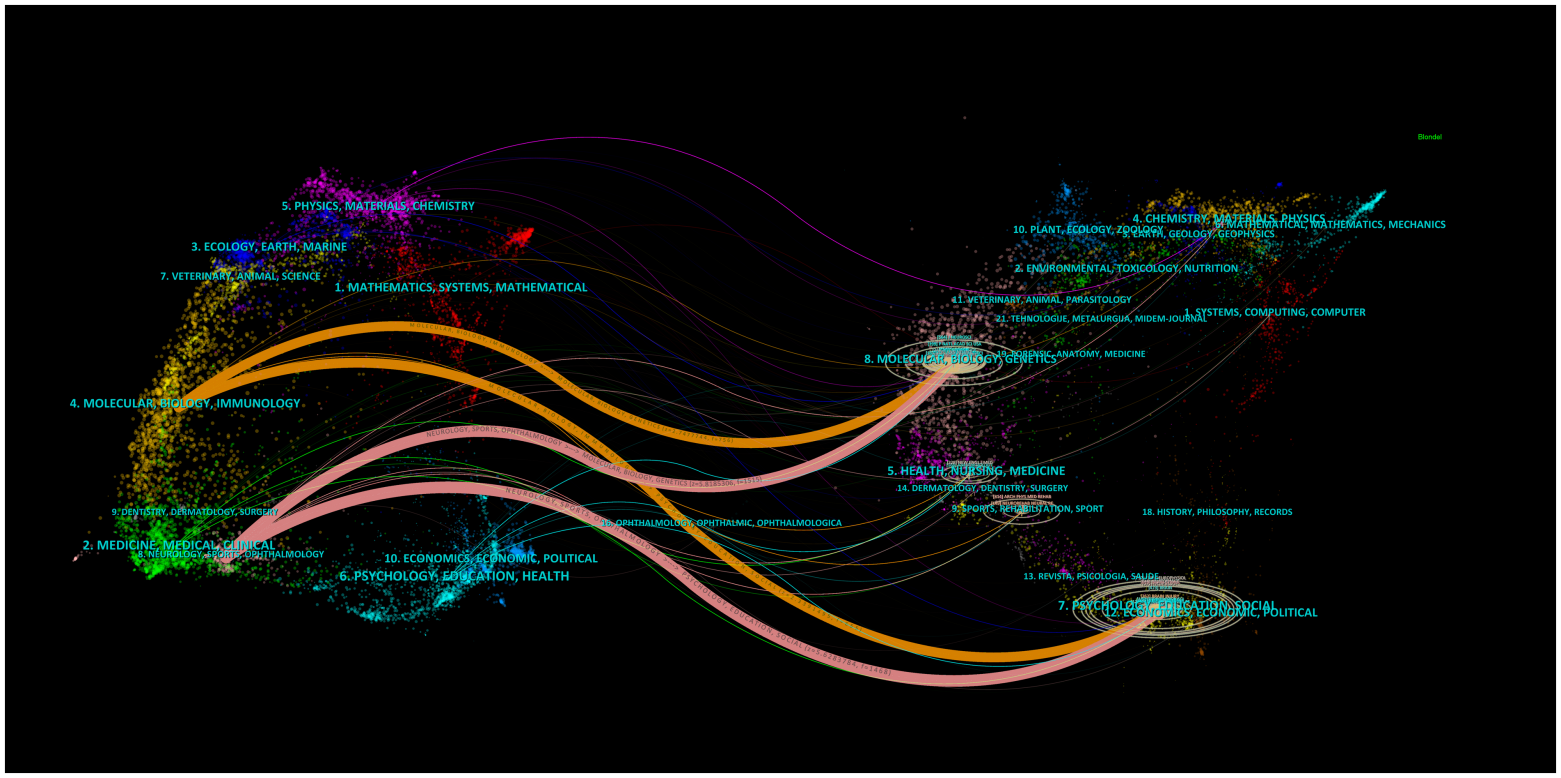
The dual-map overlay of journals in the field of neuromodulation techniques in DOC.

The dual-map overlaps of the journals are presented in Figure 4, indicating the reciprocal relationship between cited and citing journals. We identified four main citation trajectories (pink and orange), where journals in neurology, sports, and ophthalmology (pink trajectory) were significantly more frequently cited by Molecular, Biology, Genetics (*Z* = 5.82, *f* = 1,515), and Psychology, Education, Social (*Z* = 5.45, *f* = 1,468) fields. In addition, journals in Molecular, Biology, Immunology (orange trajectory) were influenced by journals in Molecular, Biology, Genetics (*Z* = 2.74, *f* = 756), and Psychology, Education, Social (*Z* = 2.34, *f* = 655) fields.

### Analysis of subject categories

**Figure 5:**
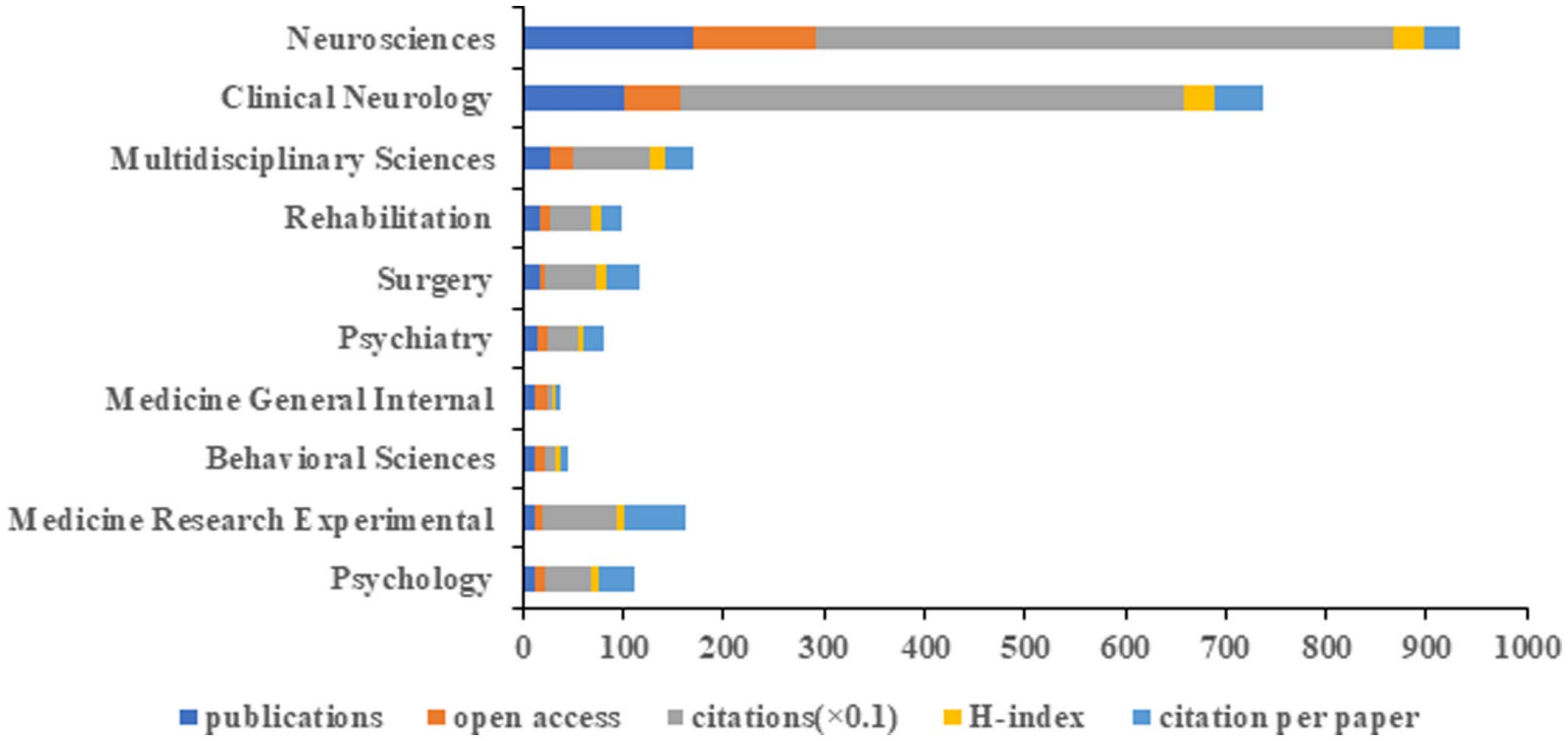
The top 10 subject categories of Web of Science in terms of publications, open-access articles, citations (×0.1), H-index, and citations per paper.

The 338 publications were sorted into 64 different WoS subject categories. We conducted an analysis of the top 10 published disciplines (Figure 5). Neurosciences led with the highest number of publications (*n* = 170), open-access value (*n* = 1,221), citations (*n* = 5,772), and H-index (*n* = 30), followed by Clinical Neurology (*n* = 100) and Multidisciplinary Sciences (*n* = 27). Medicine Research Experimental had the highestcitation per paper (*n* = 61.33).

### Analysis of references

**Figure 6:**
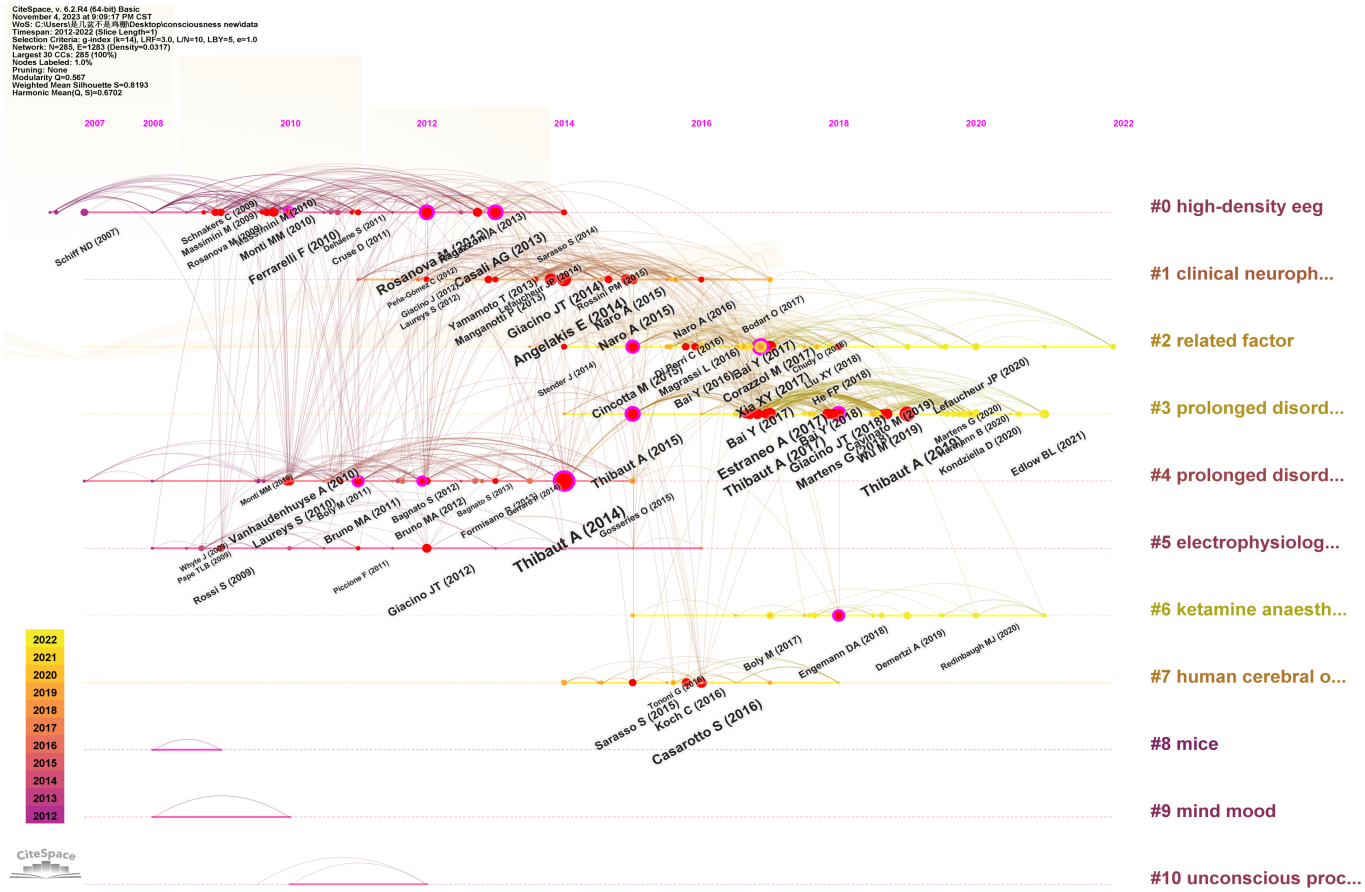
A timeline view of reference co-citation analysis.

Based on the analysis of reference co-citation, the research categories were segmented into 11 groups (#0–11). The timeline view of clusters for citation information of the cluster domains is shown in Figure 6. The largest cluster (#0) had 46 members, which was labeled as conscious state by Latent Semantic Indexing (LSI). The major citing article of the cluster was “Assessing consciousness in coma and related states using transcranial magnetic stimulation combined with electroencephalography.” The second-largest cluster (#1) had 43 members labeled as prolonged disorder by LSI. The major citing article of the cluster was “Electromagnetic brain stimulation in patients with disorders of consciousness.”

The Sigma value is calculated using a formula that combines both centrality and burstness metrics to identify innovative topics. The formula for Sigma is given as follows: Sigma = (centrality+1) burstness. We have summarized the top 3 innovative references (Table 2). Two articles were observational studies and one article was a randomized controlled trial.

**Table 2:** Three innovative studies of the neuromodulation techniques in DOC research among the cited references of the included 338 studies.

Study	Sigma*	Journal	Study type	Sample	Intervention	Outcomes	Highlights
Thibaut et al. (2014)	7.09	Neurology	Randomized Controlled Trial	55	Anodal and sham tDCS were delivered over the left DLPF cortex for 20 min in patients in VS/UWS or in MCS Neurosciences	Consciousness; CRS-R subscales; the long-term effect of tDCS.	tDCS over left DLPF cortex may transiently improve signs of consciousness in MCS following severe brain damage as measured by changes in CRS-R total scores.
Rosanova et al. (2012)	3.93	Brain	An observational study	17	NA	Transcranial magnetic stimulation combined with electroencephalography	Transcranial magnetic stimulation together with high-density electroencephalography offer an effective way to detect and track recovery of consciousness in brain-injured patients who are unable to exchange information with the external environment.
Ferrarelli et al. (2010)	2.19	Proc Natl Acad Sci U S A	An observational study	11	NA	Transcranial magnetic stimulation combined with electroencephalography	These results suggest that it might be possible to use TMS-EEG to assess consciousness during anesthesia and in pathological conditions, such as coma, vegetative state, and minimally conscious state.

^*^Sigma = (centrality+1) burstness (burstness on the index) to identify innovative reference.

### Analysis of authoritative countries, institutions, and authors

**Figure 7:**
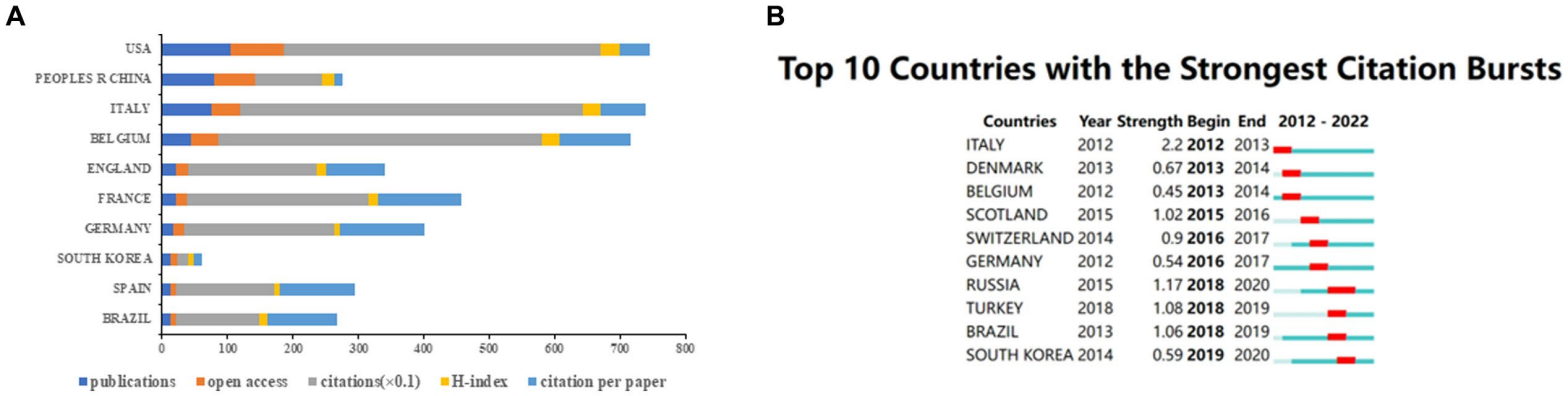
The contribution of countries of the global publications. **(A)** Top 10 countries in terms of publications, open-access articles, citations (×0.1), H-index, and citations per paper. **(B)** Top 10 countries with the highest citation bursts by CiteSpace. The blue bars indicate that the reference has been published; The red bars represent citation burstness.

Figure 7A shows the top 10 countries based on the number of publications of neuromodulation techniques in DOC research. The USA had the highest number of publications (*n* = 105), open access papers (*n* = 81), and H-index (*n* = 30). Italy had the greatest number of citations (*n* = 5,224). Germany ranked the highest number of citation per paper (*n* = 128.39). Figure 7B shows the top 10 countries with the strongest citation burst. Italy represented the strongest citation burst (strength = 2.2) from 2012 to 2013, followed by Russia (strength = 1.17) and Turkey (strength = 1.08).

**Figure 8:**
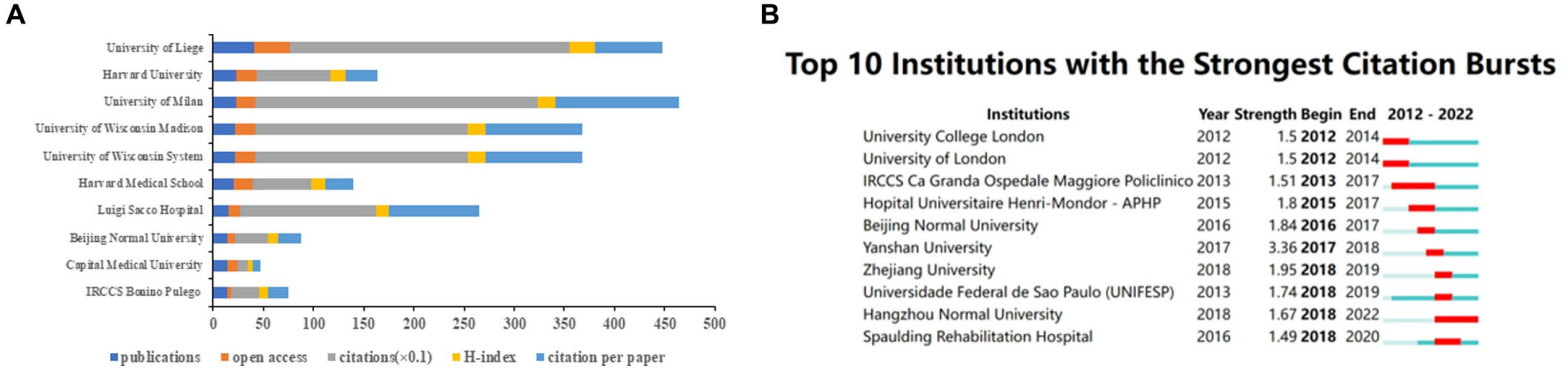
The contribution of institutions of the global publications. **(A)** Top 10 institutions in terms of publications, open-access articles, citations (×0.1), H-index, and citations per paper. **(B)** Top 10 institutions with the highest citation bursts by CiteSpace. The blue bars indicate that the reference has been published; The red bars represent citation burstness.

Figure 8A shows the top 10 institutions based on the number of publications of neuromodulation techniques in DOC research. The University of Liege has the highest number of publications (*n* = 41), open access value (*n* = 36), and H-index (25). The University of Milan ranked the highest number of citations (*n* = 2,815) and citation per paper (*n* = 122.39). Figure 8B shows the top 10 institutions with the strongest citation burst. Yanshan University represented the strongest citation burst (strength = 3.36), maintaining a high-intensity outbreak in 2017–2018.

**Figure 9:**
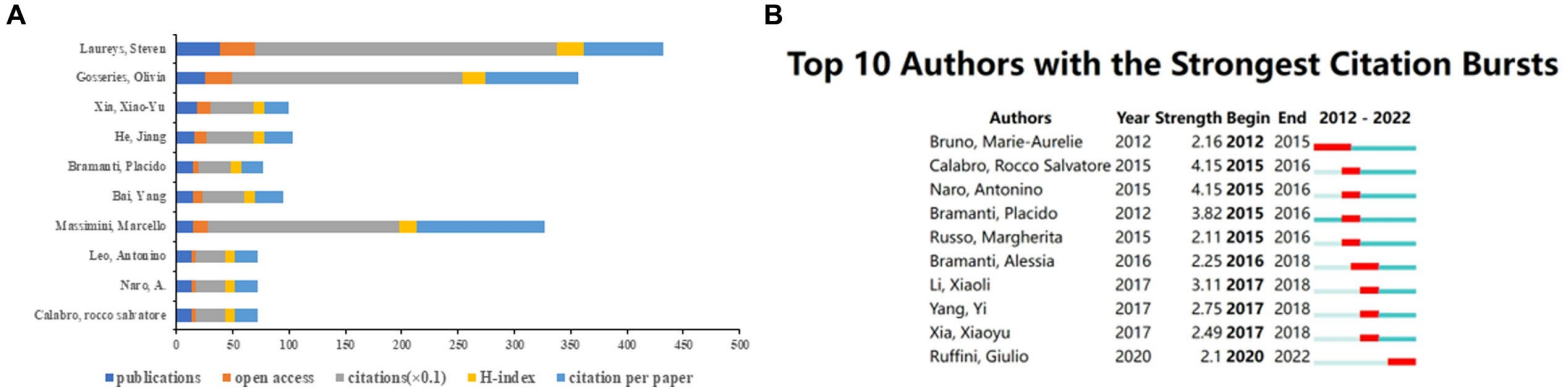
The contribution of authors of the global publications. **(A)** Top 10 authors in terms of publications, open-access articles, citations (×0.1), H-index, and citations per paper. **(B)** Top 10 authors with the highest citation bursts by CiteSpace. The blue bars indicate that the reference has been published; The red bars represent citation burstness.

Figure 9A shows the top 10 authors according to the number of publications of neuromodulation techniques in DOC research. Laureys Steven had the largest number of papers (*n* = 38), open access papers (*n* = 32), citations (*n* = 2,681), and H-index (*n* = 23). Massimini Marcello had the greatest number of citation per paper (*n* = 113.4). Figure 9B shows the top 10 authors with the strongest citation burst. Calabro, and Naro had the strongest citation burst (strength = 4.15), with the burst lasting 2 years (2015–2016).

### Analysis of coauthorship among countries, institutions, and authors.

**Figure 10:**
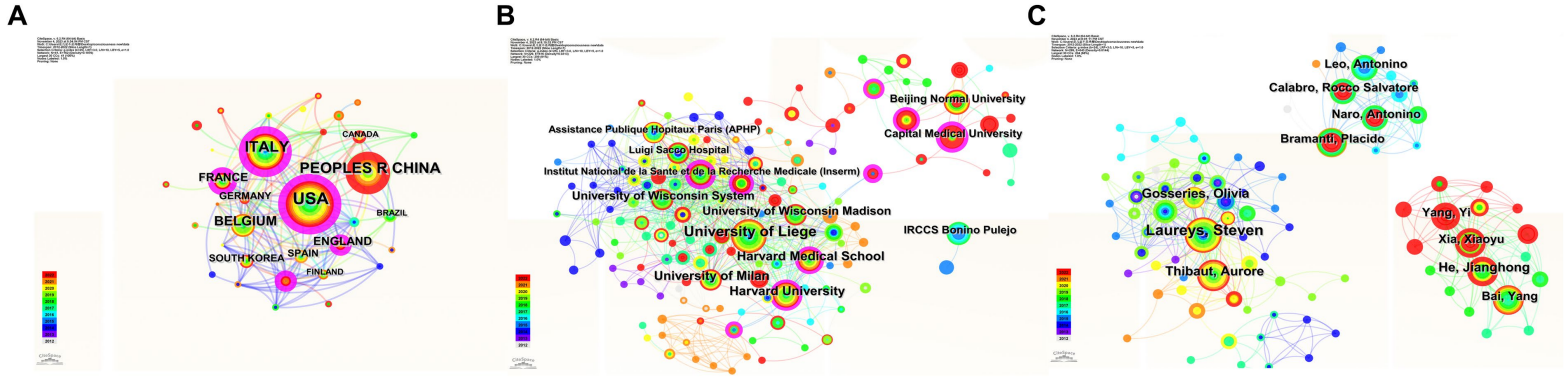
In the cooperative network analysis diagram, close internal cooperation countries **(A)**, institutions **(B)**, and authors **(C)** have been formed. The links between nodes indicate the presence of co-authorship or co-occurrence relationships.

The collaboration maps for various countries, institutions, and authors are shown in Figure 10. Regarding centrality, the top three countries were Italy (*n* = 0.32), the USA (*n* = 0.22), and England (*n* = 0.13). The top three ranked institutions by centrality were Sichuan University (*n* = 0.24), Capital Medical University (*n* = 0.23), and Institut National de la Sante et de la Recherche Medicale (*n* = 0.21). The top three centrality authors were Laureys Steven (*n* = 0.03), Ruffini Giulio (*n* = 0.02), and Rossi Simone (*n* = 0.02). Interestingly, we observed that Laureys Steven and Rossi Simone primarily conducted research on the application of transcranial magnetic stimulation together with high-density electroencephalography to assess brain connectivity in disorders of consciousness (Gosseries et al., 2014; Ragazzoni et al., 2017), while Ruffini Giuli’s investigation concentrated on examining the impact of tDCS on patients with consciousness disorders (Martens et al., 2020).

### Analysis of keywords

**Figure 11:**
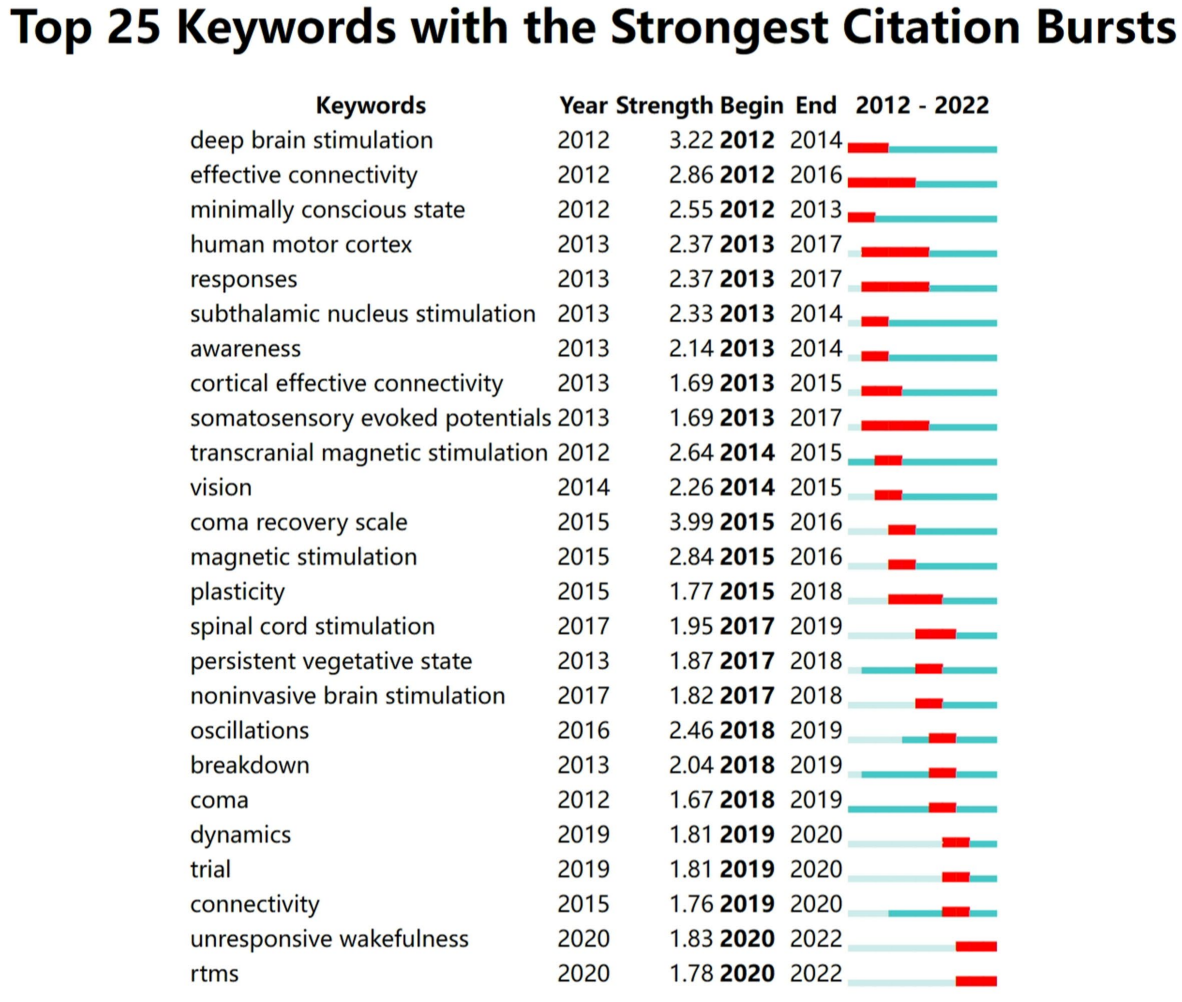
The top 25 keywords with the strongest citation bursts.

Figure 11 presents the top 25 keywords with the strongest citation bursts from 2012 to 2022. The keyword with the highest burst value was coma recovery scale (*n* = 3.99), while the keywords with the longest burst period were effective connectivity, human motor cortex, response, and somatosensory evoked potentials, lasting 5 years. At the conclusion of 2022, the most prevalent keywords among cited publications from 2020 to 2022 encompassed unresponsive wakefulness and rtms.

### Analysis of the top 10 most cited papers

The top 10 most cited papers are listed in Table 3. The citations of these articles ranged from 147 to 1,252. Three of them have been cited more than 500 times. The article entitled “Evidence-based guidelines on the therapeutic use of repetitive transcranial magnetic stimulation (rTMS)” published by Lefaucheur et al. in 2014 in Clinical Neurophysiology has been cited the most (1,252 citations) (Lefaucheur et al., 2014). Four of the top 10 articles were published in journals with an impact factor of 10 or higher, including Science Translational Medicine, Brain, Neurology, and Lancet Neurology.

**Table 3:** Top 10 most cited papers in included 338 studies.

Title	First author	Journal	Impact factor	Year	Citation (WoS)	Wos categories	Category rank
Evidence-based guidelines on the therapeutic use of repetitive transcranial magnetic stimulation (rTMS)	Lefaucheur, JP	Clinical Neurophysiology	4.7	2014	1,252	Clinical Neurology; Neurosciences	53/212; 85/272
Evidence-based guidelines on the therapeutic use of transcranial direct current stimulation (tDCS)	Lefaucheur, JP	Clinical Neurophysiology	4.7	2017	933	Clinical Neurology; Neurosciences	53/212; 85/272
A Theoretically Based Index of Consciousness Independent of Sensory Processing and Behavior	Casali, AG	Science Translational Medicine	17.1	2013	615	Cell Biology; Medicine, Research & Experimental	9/203; 4/190
Risks of common complications in deep brain stimulation surgery: management and avoidance	Fenoy, AJ	Journal of Neurosurgery	4.1	2014	276	Clinical Neurology; Surgery	63/212; 29/213
Recovery of cortical effective connectivity and recovery of consciousness in vegetative patients	Rosanova, M	Brain	14.5	2012	274	Clinical Neurology; Neurosciences	5/212; 10/272
Consciousness and Complexity during Unresponsiveness Induced by Propofol, Xenon, and Ketamine	Sarasso, S	Current Biology	9.2	2015	211	Biochemistry & Molecular Biology; Biology; Cell Biology	31/285; 5/92; 30/191
Possible mechanisms underlying the therapeutic effects of transcranial magnetic stimulation	Chervyakov, AV	Frontiers in Human Neuroscience	2.9	2015	189	Neurosciences; Psychology	174/272; 33/81
tDCS in patients with disorders of consciousness	Thibaut, A	Neurology	10.1	2014	188	Clinical Neurology	12/212
Complexity of Multi-Dimensional Spontaneous EEG Decreases during Propofol Induced General Anesthesia	Schartner, M	Plos One	3.7	2015	156	Multidisciplinary Sciences	26/73
Therapeutic interventions in patients with prolonged disorders of consciousness	Thibaut, A	Lancet Neurology	48	2019	147	Clinical Neurology	1/212

WoS, Web of Science.

## Discussion

Neuromodulatory therapies have garnered worldwide research attention over the past decade and is regarded as a potential tool that may promote neural remodeling and consciousness restoration. Additionally, neuromodulation techniques have several advantages over traditional pharmacological therapy, particularly in the use of electrical/magnetic stimulation techniques to directly regulate brain activity via either a transcranial approach or an afferent pathway. Analyzing the relevant literature on neuromodulation techniques in DOC research through bibliometric methods can assist researchers in comprehensively and systematically understanding the knowledge structure, developmental trajectory, and research hotspots in this field. Herein, we conducted a bibliometric analysis of DOC articles published from 2012 to 2022 that employed neuromodulation techniques, providing crucial insights into the current development and research frontiers of this domain.

### Global research trends of neuromodulation techniques in DOC

Based on our research results, the annual publication volume showed a continuous but irregular annual growth trend, with the most significant growth trend occurring between 2016 and 2022. However, the growth rate in Neurosciences was slower than the overall trend. Furthermore, the total number of citations for articles also showed a continuous annual growth trend, increasing from 15 to 1,781. The growth of Neurosciences was consistent with the overall growth. These results indicate that the global attention to neuromodulation techniques in DOC has been steadily increasing.

In terms of authoritative journals, Frontiers in Neurology (*n* = 16), Brain Sciences (*n* = 13), and Frontiers in Neuroscience (*n* = 13) were ranked as the top three. Among the top 10 journals, only two journals belong to Q1. Furthermore, only Brain Stimulation had an IF exceeding 5, providing a platform for high-impact neuromodulation research from an international perspective. It is thus clear that the quality of studies investigating the role of neuromodulation techniques for DOC still needs to be strengthened. In addition, by examining the dual-map overlaps of published journals to gain insights into the citation trajectories between different fields, it could be found that the neuromodulation techniques in DOC research were influenced by a wide range of fields from molecular, biology, and genetics to psychology, education, and social. These results indicate that the neuromodulation techniques in DOC research have a cross-disciplinary nature, and future research should pay more attention to the journal dynamics in these fields.

Among authoritative countries, the USA (*n* = 105) made the most contributions to this research domain, followed by China (*n* = 80) and Italy (*n* = 75). However, due to the relatively low citation frequency of most Chinese literature, the average number of citations per article was low. This phenomenon reflects the lack of overall research quality of Chinese publications and the fact that they have not yet achieved widespread dissemination in the field. As for authoritative institutions, the University of Liege ranked first (*n* = 41) in terms of the number of publications, and it is a leading European research university alliance. Additionally, this institution also exhibited the highest open-access value (*n* = 36), and H-index (*n* = 25). In the realm of authoritative authors, Laureys Steven and Gosseries Olivia stood out in this field with exceptional accomplishments, publishing over 20 papers and accumulative 2000+ citations. Moreover, Laureys Steven also exhibited the highest centrality (*n* = 0.03). From the perspective of the cooperative network, Italy had the greatest centrality (*n* = 0.32), followed by the USA (*n* = 0.22) and England (*n* = 0.13). Notably, the USA and Italy exhibited significant centrality and publication output, suggestive of occasional dual affiliations among contributors from these countries. Sichuan University (*n* = 0.24) was the top institution with the highest centrality, followed by Capital Medical University (*n* = 0.23) and Institut National de la Sante et de la Recherche Medicale (*n* = 0.21). Concurrently, the majority of the top 10 institutions with the highest centrality and publication output were from China and the USA. However, these institutions primarily collaborated with other institutions within their respective countries. It is not difficult to find that the cooperation between countries and organizations remains regional. Therefore, it is imperative to enhance collaboration and communication with domestic and foreign research institutions in the future.

### Research hotspots and frontiers of neuromodulation techniques in DOC

According to the subject categories of neuromodulation techniques in DOC research, Neurosciences (*n* = 170) ranked first in this field, followed by Clinical Neurology (*n* = 100) and Multidisciplinary Sciences (*n* = 27). The top 10 discipline categories were Neurosciences, Clinical Neurology, Multidisciplinary Sciences, Rehabilitation, Surgery, Psychiatry, Medicine General Internal, Behavioral Sciences, Medicine Research Experimental, Psychology, suggesting neuromodulation techniques for DOC are a complex medical challenge that necessitates multidisciplinary communication and cooperation.

In terms of reference analysis, the earliest of the 10 most cited articles was published in 2012, while the latest was published in 2019, where, BRAIN (Rosanova et al., 2012) and, LANCET NEUROL (Thibaut et al., 2019) were defined as classic literature by the bibliometric method, highlighted their significant academic value in this field. Additionally, the most relevant citer to the largest cluster was “Assessing consciousness in coma and related states using transcranial magnetic stimulation combined with electroencephalography.” This review pointed out that TMS-EEG has great potential in identifying consciousness markers at the individual level, and may be of great value for clinicians in assessing consciousness (Gosseries et al., 2014). Moreover, through examination of the three innovative studies, by Thibaut et al. (2014), Rosanova et al. (2012) and Ferrarelli et al. (2010), it was found that NINT, especially TMS and tDCS, played a significant role in the evaluation and management of DOC. NINT seems to have more advantages in treating disorders of consciousness, but the reasons are complex. INT often requires surgery or implantable devices, which face the risks of infection, surgery, and device failure (Zaghi et al., 2009). NINT relies on external stimuli to regulate the nerves, which is subject to the accuracy of the device, operating skills, and the selection of stimulation parameters. In human studies, ethical considerations are also important, as INT must comply with medical ethics and fully consider potential conflicts of interest (Giacino et al., 2012), such as the relationship between researchers and device manufacturers. Future research should explore various electrophysiological assessments and treatments that offer new possibilities for enhancing the quality of life of patients with DOC.

In the domain of bibliometrics, keywords hold a significant position in summarizing articles. Through keyword analysis, we can deeply explore and reveal emerging research trends, and provide targeted guidance for future research. “coma recovery scale” was the keyword with the highest burst value, and was also the core content of assessment in DOC. Furthermore, in terms of count numbers and centrality, the top three keywords were UWS/VS, TMS and MCS. In addition, the keywords with the most outbreaks of cited publications included unresponsive wakefulness and rTMS by the end of 2022. Based on the above results, “UWS/VS,” “MCS” and “TMS” indicate potential research hotspots and frontiers. We now proceed to a detailed examination of these potential hotspot terms.

#### UWS/VS

UWS/VS refers to awake patients with brain injury who appear to lack awareness. In terms of detection and prediction, recent studies showed that neuromodulation techniques combined with electroencephalography could accurately estimate the neurobehavioral progression of UWS/VS patients for predicting their most likely clinical course and guiding clinical decisions (Arai et al., 2021; Liu et al., 2022). Besides, the treatment of UWS/VS patients remains a challenge for clinicians due to the current absence of evidence-based treatment guideline. Current studies seem to support the therapeutic effectiveness of DBS and NINT on consciousness in these patients (Angelakis et al., 2014; Naro et al., 2015; Gottshall et al., 2019; Osińska et al., 2022). However, while NINT was non-invasive and easy-to-operate, DBS had the potential to cause significant side effects. Compared with DBS, surgical techniques are less invasive, such as sVNS (Corazzol et al., 2017), which might offer a balance between efficacy and invasiveness, but further research is needed. Thus, further research involving high-quality methods and large samples of neuromodulation technology for UWS/VS is needed.

#### MCS

MCS is a transitional stage of UWS/VS, manifested as potential behavioral signs, including visual signs, motor signs, auditory localization, and habituation of auditory startle reflex (Hermann et al., 2020; Martens et al., 2020; Noé et al., 2021). In the invasive brain stimulation, only Schiff et al. demonstrated the efficacy of DBS in this particular patient through strictly design (Schiff et al., 2007). On the contrary, recent systematic review showed that NINT appeared to be effective interventions for improving MCS patients (Feng et al., 2020; Liu et al., 2023). In particular, tDCS has been shown through well-designed studies to have a limited capacity for enhancing functional recovery (Thibaut et al., 2014; Martens et al., 2018). With such progress, NINT will continue to be the important driving force for the development of patients with MCS.

#### TMS

Over the past decade, TMS has been increasingly utilized in clinical settings to treat patients with DOC, given that it is a non-invasive and non-painful brain stimulation device capable of modulating brain excitability and neural networks. As early as 2009, Louise-Bender Pape et al. (2009) found that DOC patients tended to show significant neurobehavioral improvements that were temporally associated with the delivery of rTMS. Recently, Huang et al. (2023) reported the efficacy and possible mechanisms of rTMS for DOC and discussed the potential key parameters that affected the therapeutic efficiency of rTMS in DOC patients. Additionally, iTBS was a novel rTMS model that may be suggested as a potential therapeutic intervention of DOC (Wu et al., 2018). However, the committee did not recommend any level of evidence or use of TMS for chronic DOC patients (Lefaucheur et al., 2020), indicating the problems of small sample size and lack of high-quality research in these patients. Therefore, it is necessary to further study and develop the most effective treatment plan for DOC patients by determining the optimal stimulation strategy.

## Strengths and limitations

This study uses CiteSpace software to conduct a bibliometric analysis of literature concerning neuromodulation techniques in DOC for the first time, and our results provide an in-depth analysis of this field from multiple perspectives, including knowledge structure, development trajectory, and research hotspots, which could help researchers in conducting profound investigations into this emergent and promising field of study. Nevertheless, there are certain limitations to this study. Firstly, the CiteSpace software has certain limitations, which results in the literature data being solely derived from one database (WoSCC). Additionally, some important studies may be excluded because of the omission of literature in non-English languages. Finally, the incomplete dataset of this year inevitably limits its consideration in this research.

## Conclusion

This bibliometric study may provide investigators with a fresh perspective on the current development and emerging trends of neuromodulation techniques in DOC from 2012 to 2022. The most influential journal, country, institution, and author were Frontiers in Neurology, the USA, the University of Liege, and Laureys Steven. By combining the reference and keyword analysis, we can deduce that the accurate diagnosis of consciousness levels in patients with DOC and the exploration of effective neuromodulation-based assessment and treatment methods for this population are currently at the forefront of research in this field. For instance, the combination of TMS and EEG may hold significant potential in the assessment of DOC. Moreover, NINT, particularly, are anticipated to emerge as prominent therapeutic modalities in the forthcoming era for the management of patients afflicted with consciousness disorders. There is no doubt that the domain of neuromodulation techniques in DOC will continue to attract an increasing number of researchers, leading to more valuable and meaningful research for the benefit of patients.

## Data availability statement

The original contributions presented in the study are included in the article/supplementary material, further inquiries can be directed to the corresponding authors.

## Author contributions

BG: Data curation, Writing – original draft. QH: Data curation, Writing – original draft. JN: Writing – original draft. ZY: Conceptualization, Writing – review & editing.
